# Ischemic Preconditioning Increases Endothelial Progenitor Cell Number to Attenuate Partial Nephrectomy-Induced Ischemia/Reperfusion Injury

**DOI:** 10.1371/journal.pone.0055389

**Published:** 2013-01-31

**Authors:** Hao Liu, Ran Wu, Rui-Peng Jia, Bing Zhong, Jia-Geng Zhu, Peng Yu, Yan Zhao, Yu-Zheng Ge, Jian-Ping Wu

**Affiliations:** Department of Urology, Nanjing First Hospital, Nanjing Medical University, Nanjing, China; UCL Institute of Child Health, United Kingdom

## Abstract

**Objectives:**

The objective of this study was to investigate the role of endothelial progenitor cells (EPCs) in the modulation of ischemia-reperfusion injury (IRI) in a partial nephrectomy (PN) rat model using early-phase ischemic preconditioning (IPC).

**Materials and Methods:**

Ninety male Sprague-Dawley rats were randomly divided into three groups following right-side nephrectomy: Sham-operated rats (surgery without vascular clamping); PN rats (renal blood vessels were clamped for 40 min and PN was performed); and IPC rats (pretreated with 15 min ischemia and 10 min reperfusion). At 1, 3, 6, 12, 24 h, and 3 days after reperfusion, the pool of circulating EPCs and kidneys were harvested. The extent of renal injury was assessed, along with EPC number, cell proliferation, angiogenesis, and vascular growth factor expression.

**Results:**

Pretreated rats exhibited significant improvements in renal function and morphology. EPC numbers in the kidneys were increased at 12 h following reperfusion in the IPC group as compared to the PN or Sham groups. Cell proliferation (including endothelial and tubular epithelial cells) and angiogenesis in peritubular capillaries were markedly increased in kidneys treated with IPC. In addition, vascular endothelial growth factor-A (VEGF-A) and stromal cell-derived factor-1α (SDF-1α) expression in the kidneys of pretreated rats was increased compared to rats subjected to PN.

**Conclusions:**

Our investigation suggested that: (1) the early phase of IPC may attenuate renal IRI induced by PN; (2) EPCs play an important role in renal protection, involving promotion of cell proliferation and angiogenesis through release of several angiogenic factors.

## Introduction

Partial nephrectomy (PN) exhibits similar efficacy in treating renal cancers as radical nephrectomy (RN) and is superior to RN in preserving renal function and prevention of chronic kidney disease **[1]**–**[6]**. However, renal hilar clamping causes warm ischemia (WI), with the potential for renal ischemia/reperfusion injury (IRI) [7], [8]. It has been recently demonstrated that endothelial progenitor cells (EPCs) contribute to the restoration of renal function after IRI. EPC transplantation was associated with improvement in renal function following IRI, and has been explained by enhanced repair of renal microvasculature, tubule epithelial cells and synthesis of high-levels of pro-angiogenic cytokines, which promoted proliferation of both endothelial and epithelial cells [9]. Moreover, EPC incompetence may be an important mechanism of accelerated vascular injury and eventually lead to chronic renal failure [10]. However, the number of EPCs in the circulation and bone marrow of adults is insufficient to repair IRI in affected organs [11] and the number of EPCs that can be transplanted into the circulation is limited. Hence, the ability to sufficiently increase the number of EPCs has become an issue of concern.

Studies have confirmed that ischemic preconditioning (IPC) is an innate phenomenon in which brief exposure to sublethal ischemia induces a tolerance to injurious effects of prolonged ischemia in various organs [12] and is also an effective method to increase the number of EPCs [13], [14]. IPC has two distinct phases: The early phase of IPC is established within minutes and may last for several hours. Conversely, the late phase of protection requires hours to days to develop and becomes apparent after 24 h to several days [13], [15]. However, the interval between pre-ischemic and ischemic injury is too long for clinical application. Hence, we focused on the early phase of IPC in this study.

Li et al. [14] investigated whether the early phase of IPC could produce rapid increases in the number of circulating EPCs in the myocardium, with the goal of directly preserving the microcirculation in the ischemic myocardium by incorporation of EPCs into vascular structures. They also assessed whether EPCs could act as vascular endothelial growth factor (VEGF) donors in ischemic myocardium. Therefore, it appears logical to determine whether the early phase of IPC could protect the remaining renal tissue following PN through the mechanism described above. Thus, the present study was designed to investigate the effects of IPC on renal IRI induced by PN, as well as the possible mechanisms in an experimental model of PN.

## Materials and Methods

### Animals and Surgical Procedures

Ninety male Sprague-Dawley rats weighing 250–300 g and aged 2–3 months were bred and housed in the animal house of the Experimental Animal Centre affiliated with Nanjing First Hospital. The rats were housed in individual cages at 20–25°C with a 12 h: 12 h light-dark cycle, and fed standard laboratory chow and tap water *ad libitum*, but were fasted for 24 h prior to surgery. All animal procedures were approved by the Committee on the Ethics of Animal Experiments of Nanjing Medical University.

All rats were anaesthetized using sodium pentobarbital (50 mg/kg i.p.) and placed on a warming table to maintain a rectal temperature of 37°C. A transverse 1 cm lumbotomy incision was performed and the rats were randomly divided into three groups of 30 animals following right-side nephrectomy. For the sham-operated group, the left renal artery was separated without clamping of the renal artery. For the PN group, the left kidney was isolated from the abdomen, the renal pedicle was blocked with a non-traumatic vascular clamp for 40 min while a lower pole PN was performed, and the kidney was covered using a piece of gauze soaked with warm isotonic saline (37°C). For the IPC group, the left renal artery was blocked for 15 min, and then reperfused for 10 min before a 40-min occlusion and PN.

The rats were anesthetized again using sodium pentobarbital (i.p.) to harvest the pool of circulating EPCs and to sample the left kidney at 1, 3, 6, 12, 24 h and 3 days following reperfusion (each group contained five rats per time point). The abdomen was opened and the left kidney was perfused with PBS and then rapidly removed; one third of each kidney was fixed in 4% formalin to assay the extent of renal injury and EPC number as well as cell proliferation and angiogenesis in peritubular capillaries. One-third of the kidney was saved on ice for monoplast suspensions and the residual kidney was rapidly frozen in liquid N_ 2_, and stored at −80°C for the detection of vascular growth factor expression.

### Biochemical Examination

Blood (2 ml) was obtained from the inferior vena cava. Samples were centrifuged at 2000 g for 10 min and the supernatants were collected to measure serum levels of BUN and creatinine (SCr) using clinically automated analysis methods (Hitachi 7600-10, Hitachi High-Technologies Corporation, Japan).

### Histological Examination

Formalin-fixed tissues were embedded in paraffin and sectioned at 5 µm and stained with haematoxylin and eosin (H&E). The sections were examined microscopically by an experienced pathologist who was blinded to the treatment that each animal received. Renal injury was defined as tubular necrosis, tubular dilatation and/or atrophy, inflammatory cell infiltration, cellular edema, or tubule cast formation [16], with a scoring range from Grade 0 to 4. Higher scores represent more severe damage: 0, normal kidney; 1, minimal necrosis (<25% involvement of the cortex or outer medulla); 2, mild necrosis (25–50% involvement of the cortex or medulla); 3, moderate necrosis (50–75% involvement of the cortex or medulla); and 4, severe necrosis (>75% involvement of the cortex or medulla).

### Immunohistochemical Staining

Peritubular capillary rarefaction index (PCRI) was analyzed for peritubular capillary densities using a monoclonal antibody to CD34 and stained using immunohistochemistry. To determine cell proliferation, immunohistochemical staining with proliferating cell nuclear antigen (PCNA) was performed. Briefly, paraffin-embedded blocks were sectioned at a 5 µm, dewaxed and rehydrated. Antigens were retrieved with microwave pretreatment in citrate buffer (pH 6.0). Endogenous peroxidase was blocked with 3% H_2_O_2_ for 15 min, and then nonspecific binding sites were blocked with 4% goat serum diluted 1∶10 in PBST (PBS, pH 7.4, 0.05% Tween 20). Sections were incubated with a rabbit anti-CD34 antibody (ABbiotec, San Diego, CA, USA) at 1∶100 dilution or rabbit anti-PCNA antibody (Santa Cruz Biotechnology, Santa Cruz, CA, USA) at 1∶100 dilution overnight at 4°C. Primary antibodies were detected with horseradish peroxidase-conjugated goat anti-rabbit secondary antibody and developed with 3,3′-diaminobenzidine tetrahydrochloride. Negative controls were performed by omitting the primary antibody. Finally, PCRI was determined using the procedure of Kang et al [17], and the number of PCNA-positive cells were counted in 10 non-overlapping sequential fields at 400×magnification [18].

### Immunofluorescence Staining

To observe the number of EPCs residing in the kidney, double immunofluorescence staining was performed. Briefly, deparaffinized tissue sections were blocked with 4% goat serum to decrease nonspecific staining and then incubated with 1∶100 rabbit anti-CD34 antibody (ABbiotec) and 1∶100 mouse anti-Flk antibody (Santa Cruz Biotechnology) at 4°C overnight. Primary antibody was detected using PE-conjugated goat anti-rabbit and FITC-conjugated goat anti-mouse secondary antibody for 2 h in the dark. Finally, CD34^+^/Flk^+^ protein expression was analyzed and evaluated with confocal laser scanning microscopy.

### Flow Cytometry

For quantification of EPCs in ischemic kidneys using flow cytometry, mononuclear cells were isolated by a mechanical process. Kidney tissue (about 100 mg) was homogenized with 2 ml PBS in a glass homogenizer and then filtered through a 200 µm nylon mesh (BD Biosciences; San Jose, CA, USA). Single-cell suspensions were centrifuged at 2000 g for 5 min, resuspended with 2 ml PBS, and then incubated for 30 min with a rabbit polyclonal anti-Flk-1 antibody (Novus Biologicals; Littleton, CO, USA) and FITC-conjugated mouse monoclonal anti-CD34 antibody (Santa Cruz Biotechnology) at room temperature. The secondary antibody for Flk-1 staining was goat anti-rabbit IgG-PE (Santa Cruz Biotechnology). After washing, immunofluorescence was detected using flow cytometry. To quantify EPCs, the number of CD34/Flk-1 double-positive cells within the mononuclear cell population was counted.

### q-PCR Analysis

mRNA expression of VEGF-A, stromal cell-derived factor-1α (SDF-1α) and insulin like growth factor 1 (IGF-1) was assessed using qPCR. Total RNA was extracted from renal tissue with Trizol reagent (Invitrogen; Carlsbad, CA, USA). Total RNA concentration was measured using UV spectrophotometry at 260 nm, purity was determined by the 260 nm/280 nm absorbance ratio, and quality was confirmed using agarose gel electrophoresis. Total RNA (1 µg) was reverse transcribed using oligo dT primers and Reverse Transcription System (Promega; Madison, WI, USA) according to the manufacturer’s instructions. Transcript expression was analyzed using qPCR with a 7500 Real-Time PCR System (Applied Biosystems; Carlsbad, CA, USA) and GoTaq qPCR Master Mix (Promega) as the fluorophore. PCR was performed in triplicate for 40 cycles comprising an initial denaturation stage of 95°C for 2 min, followed by 95°C for 15 s and finally 60°C for 1 min. Primers were designed using Primer Express 2.0 (Applied Biosystems) as follows: glyceraldehyde-3-phosphate dehydrogenase (GAPDH)– F5′-TGTCGTGGAGTCTACTGGCGTCTT-3′, R5′-GAGGGAGTTGTCATATTTCTCGTGGT-3′; VEGF– F5′-CGAGACGCAGCGACAAGGCA-3′, R5′-ACCTCTCCAAACCGTTGGCACG-3′; SDF-1– F5′-GAGCCATGTCGCCAGAGCCAAC-3′, R5′-CACACCTCTCACATCTTGAGCCTCT-3′; IGF-1– F5′-TTACTTCAACAAGCCCACAGG-3′, R5′-TACATCTCCAGCCTCCTCAGA-3′. A standard curve was constructed in each of the experimental repetitions by serial dilution of cDNA (1 to 1∶10000). PCR specificity was examined by dissociation curve analyses. To determine the relative concentration of the products, we used the comparative C_T_ (2^−ΔΔC^_T_) method, according to the instructions supplied by Applied Biosystems.

### Western Blotting Analyses

Total proteins were extracted from kidney tissues with a protein extraction kit (KeyGEN Biotechnology; Nanjing, China). SDS-PAGE and immunoblotting were performed according to the manufacturer’s instructions (Bio-Rad; Hercules, CA, US). Briefly, polyvinylidene fluoride membranes (Millipore; Billerica, MA, USA) were blocked with 5% skim milk in Tris-buffered saline (pH 7.5)-Tween 20 for 2 h at room temperature after protein transfer from 12% SDS-PAGE gels (50 µg/lane), and the membranes were subsequently incubated overnight at 4°C with either rabbit polyclonal anti-VEGF antibody (1∶1000; Santa Cruz Biotechnology), rabbit polyclonal anti-β-actin antibody (1∶2000; BOIS; Beijing, China), rabbit polyclonal anti-SDF antibody (1∶1000; Santa Cruz Biotechnology), or rabbit polyclonal anti-IGF-1 antibody (1∶1000; Abcam; Cambridge, MA, USA). The following day, membranes were extensively washing with TBST buffer and incubated with horseradish peroxidase conjugated anti-rabbit secondary antibody (KeyGEN Biotechnology) for 2 h, developed with an enhanced chemiluminescence system (ECL kit; KeyGEN Biotechnology), and images were then captured on light-sensitive imaging film.

### Statistical Analyses

All data are expressed as mean ± SEM. The means of the different groups were compared using one-way analysis of variance (ANOVA). The level of significance for all comparisons was set at *P*<0.05 or 0.01.

## Results

### Biochemical Examination

**Figure 1:**
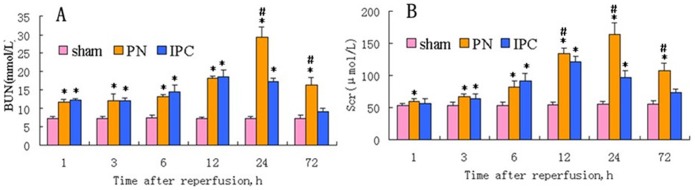
Time-dependent changes in renal function in the treatment groups. A. BUN (mmol/L); **B.** SCr (µmol/L). Each histogram represents mean ± SEM. *Significant difference vs. Sham group (*P*<0.05); ^#^significant difference vs. IPC group (*P*<0.05).

There were significant increases in SCr and BUN in the PN and IPC groups compared to the Sham group, with the exception of BUN in the IPC group at 72 h and SCr in the IPC group at 1 h and 72 h. SCr and BUN levels decreased in the IPC group as compared to the PN group at 12–72 h and 24–72 h, respectively (*P*<0.05) (Fig. 1).

### Renal Tubular Injury

**Figure 2:**
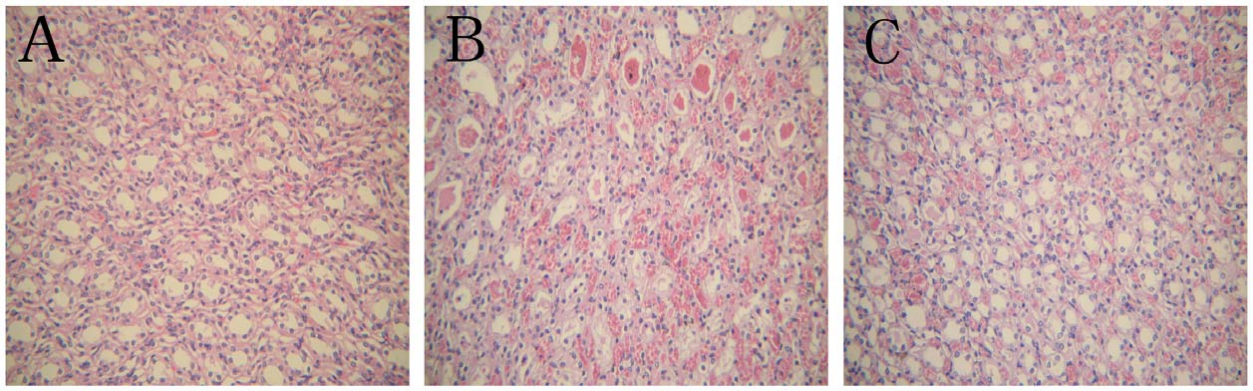
Renal tissue histological examination at 24 h following reperfusion. Renal sections were stained with hematoxylin and eosin and examined using light microscopy at a magnification×200. **A.** Sham rats exhibited minimal pathological changes in the kidneys. **B.** Following PN, more severe lesions were observed in renal tubules, with tubular atrophy, dilatation, and intratubular casts, as well as congestion in the peritubular capillaries, massive epithelial cells, atrophic epithelial lining, and intraluminal necrotic debris. **C.** IPC caused a significant reduction in the severity of acute tubular necrosis.

As demonstrated in Table 1, histological score was significantly increased in the IPC and PN groups compared to the Sham group at all time points following reperfusion (*P*<0.05). The histological score in the IPC group was decreased compared to the PN group at 12 h and 24 h (*P*<0.05). Light microscopic examination identified acute tubular necrosis in the PN group in the form of marked dilatation and/or atrophy, massive epithelial cells, atrophic epithelial lining, pyknotic nuclei, intraluminal necrotic debris, tubule cast formation, and congestion in the peritubular capillaries, especially at 24 h. These findings were much less pronounced in those kidneys treated with IPC (Fig. 2).

**Table 1:** Histopathologic scores in the three treatment groups at various time-points.

	1 h	3 h	6 h	12 h	24 h	72 h
Sham	0	0	0	0.20±0.45	0	0
PN	1.40±0.55*	0.80±0.84*	2.00±0.71*	3.00±0.71*	3.60±0.55*	3.00±0.71*
IPC	1.20±0.45*	1.00±0.71*	1.80±0.84*	1.80±0.45*#	2.60±0.55*#	2.20±0.45*

All data are expressed as mean ± SD. *P<0.05, vs. sham group. #*P*<0.05, vs. PN group.

### Effects of IPC on Accumulation of EPCs in the Kidney

**Figure 3:**
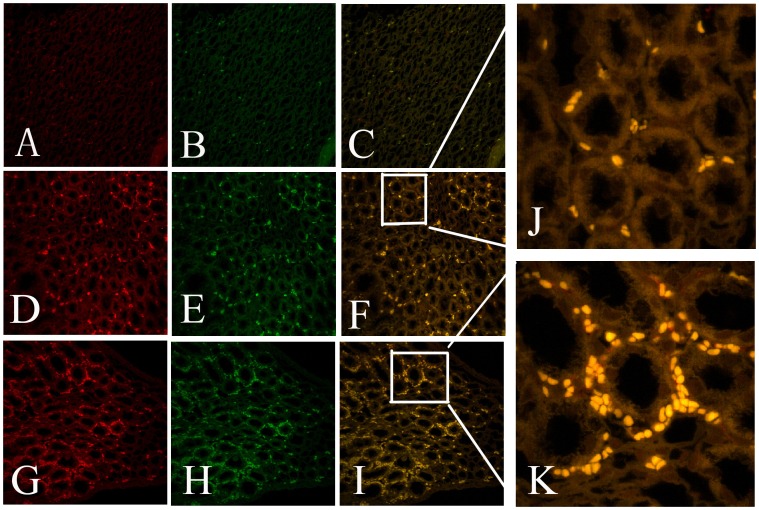
Double immunofluorescence staining to show the presence of EPCs in the ischemic kidney at 12 h after reperfusion. Cellular co-expression of CD34 (red) and flk (green) in kidney medulla indicating the presence of EPCs. In Sham tissues (**A–C**), there was no or only slight expression of EPCs. PN (**D–F**) caused higher fluorescence intensity of EPCs in renal medulla. IPC (**G–I**) caused significant increases in the intensity of fluorescence of EPCs. [Magnification: ×200 (A–I), ×400 (J, K)].

**Figure 4:**
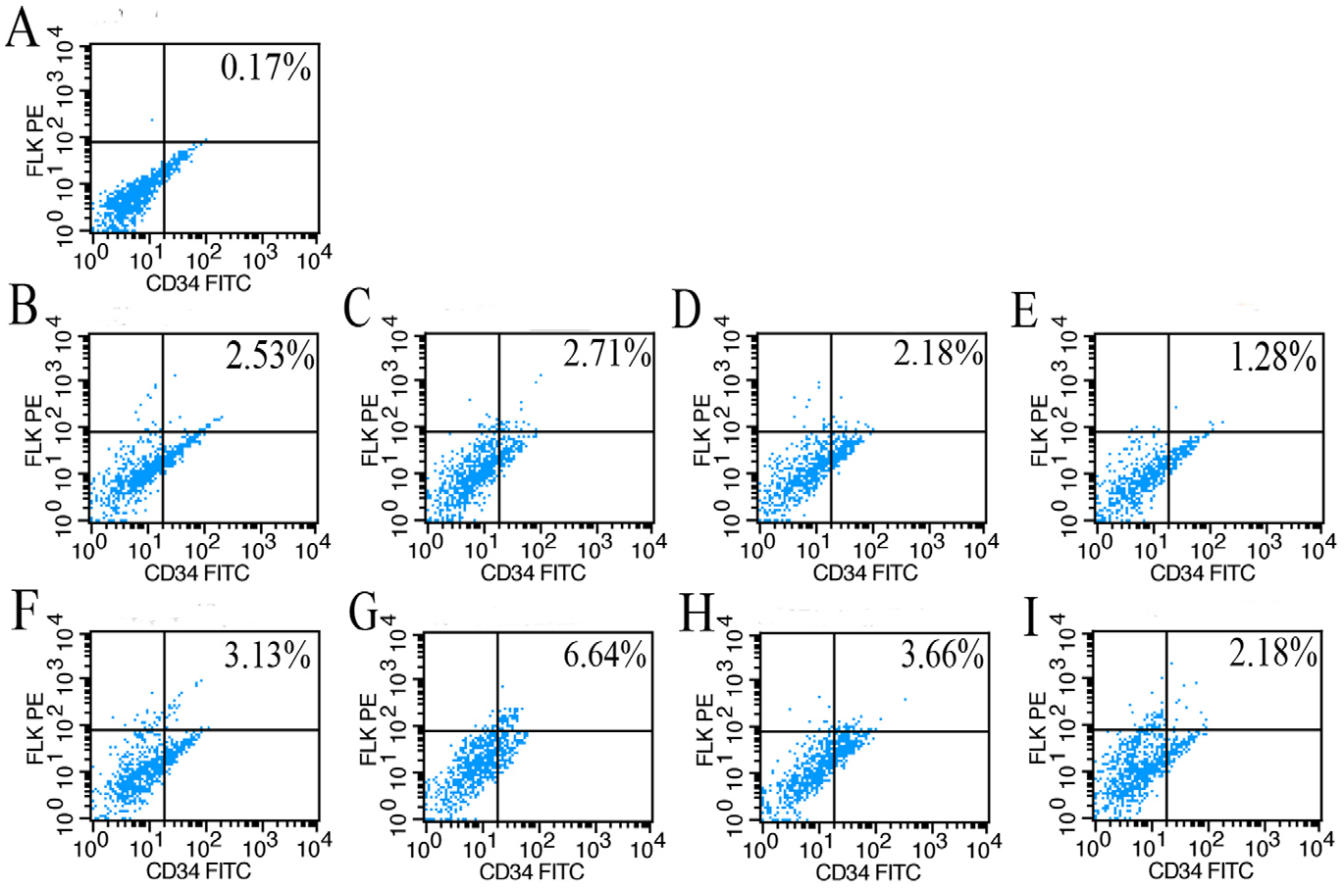
Quantitative evaluation of endothelial progenitor cells (EPCs) in kidney by FACS analyses. Representative FACS data, in which the CD34^+^/Flk-1^+^ cells from the PN group (**B–E**) and IPC group (**F–I**) were judged as EPCs. Analyses of kidney samples were performed at various time points [1 h, 6 h (not shown), 3 h (B, F), 12 h (C, G), 24 h (D, H) and 72 h (E, I) after release of the clamp; Sham group (A)].

**Figure 5:**
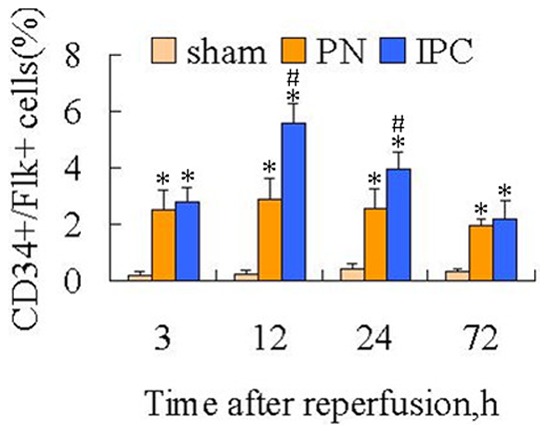
Percentage of CD34^+^/Flk-1^+^ cells within the kidney mononuclear cell population. In the PN group, the percentages of EPCs within the kidney mononuclear cell population were not significantly different following renal reperfusion at any of the time points. In contrast, treatment with IPC resulted in a marked increase in EPC number. Data are shown as mean ± SEM. *Significant difference vs. Sham group (*P*<0.05); ^#^significant difference vs. PN group (*P*<0.05).

Immunofluorescence analyses and flow cytometry were performed to elucidate whether the differences in function and morphology of the kidneys between the PN and IPC groups were associated with increases in the number of EPCs in the ischemic organ. An immunofluorescence assay was used to observe the precise location of EPCs in the kidney. EPCs were detected in tissues using double staining with antibodies to CD34 and flk. CD34^+^/flk^+^ cells were mainly concentrated in the renal medulla, particularly in the medullopapillary region, but only a modest representation was observed in the cortex of kidneys from any of the experimental groups. In addition, in the medullopapillary parenchyma, the number of double-positive cells was significantly higher in preconditioned rats compared with non-preconditioned animals. In renal tissues from Sham rats, however, there was rare expression of CD34^+^/Flk^+^ cells in renal tubular cells (Fig. 3). For quantitation of EPCs in ischemic kidneys, flow cytometry was performed. The percentage of double-positive cells was increased in the IPC and PN groups at all time points compared to controls (*P*<0.05). It is worth noting that the number of EPCs was increased at 12 h and 24 h following reperfusion compared with the PN group. These results suggested that IPC could increase the number of EPCs in the renal medullopapillary region (Fig. 4, Fig. 5).

### Cell Proliferation and Neovascularization

**Figure 6:**
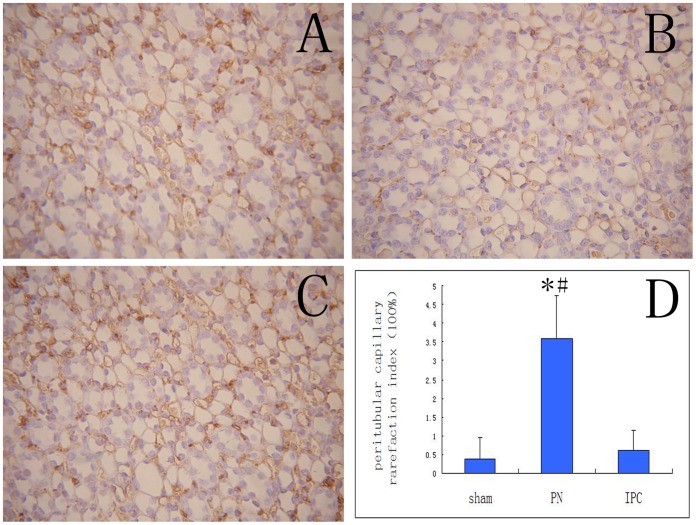
Immunohistochemical staining for CD34 at 24 h after reperfusion (×200). CD34 expression was decreased in PN group (**B**) compared with the IPC group (**C**) and the Sham group (**A**). PCRI in the PN group was significantly increased compared to the IPC group and the Sham group (*P*<0.05), however, there was no significant difference between the Sham and IPC groups. Data are shown as mean ± SEM (**D**). *Significant difference vs. Sham group (*P*<0.05); ^#^significant difference vs. IPC group (*P*<0.05).

CD34 immunochemistry was used to investigate whether attenuation of renal injury in the IPC group was associated with angiogenesis promoted by EPCs. We detected the most significant effect of IPC at 24 h after reperfusion (Fig. 6). Peritubular capillary density in the PN group was significantly reduced compared to that in the IPC and Sham groups (*P*<0.05). However, there was no significant difference between density in the Sham and IPC groups. The PCRI was 0.60±0.55% in rats with IPC, 3.60±1.14% in PN samples, and 0.40±0.55% in the Sham group.

**Figure 7:**
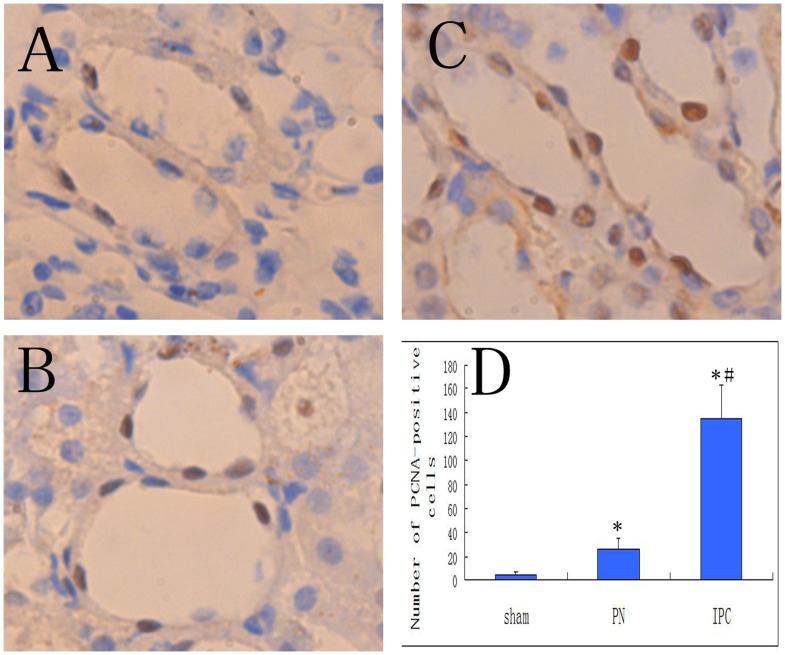
Cell proliferation in three groups at 24h after reperfusion as shown by expression of PCNA in the renal medulla. (Magnification: ×1000). In Sham tissues (A), there was no or only slight minimal proliferation. PN (B) caused higher proliferation in the renal medulla. IPC (C) caused significantly stronger staining for PCNA. The number of PCNA-positive cells was increased in the PN group when compared to the Sham group, and the IPC group showed a greater increase in PCNA-positive cells when compared to the PN group. Data are shown as mean ± SEM (D). *Significant difference vs. Sham group (*P*<0.05); ^#^significant difference vs. PN group (*P*<0.05).

To assess the number of proliferating cells, immunochemical staining with PCNA was performed. The most significant effect of IPC was detected after 24 h of reperfusion. As depicted in Fig. 7, the Sham group exhibited a minimal degree of cell proliferation as evaluated using PCNA staining. IPC treatment significantly promoted cell proliferation compared with the PN group, as reflected by the number of PCNA-positive cells (135±28% vs. 26.0±9.1%, *P*<0.05). The majority of the proliferating cells were capillary endothelial cells while a minority were renal tubular epithelial cells. This might be related to the effects on EPCs, which accumulated in ischemic kidneys, and are mediated by IPC.

### mRNA Expression of Angiogenic Factors

**Figure 8:**
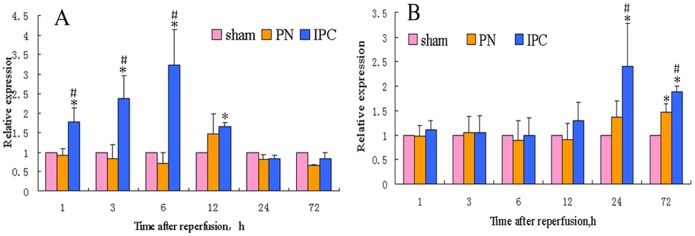
Relative expression of VEGF-A (A) and SDF-1α (B) mRNA. *Significant difference vs. Sham group (*P*<0.05); ^#^significant difference vs. PN group (*P*<0.05).

qPCR was used to investigate the levels of mRNA of angiogenic factors in the kidney. VEGF-A mRNA expression was significantly higher in IPC rats compared with the other two groups in the early phase following reperfusion (1–6 h) (*P*<0.05), but was not detected after 12 h. When investigating mRNA levels of SDF-1α, a significantly increased SDF-1α expression was observed in the PN group at 72 h and in the IPC group at 24–72 h compared to the Sham group (*P*<0.05). Further, SDF-1α mRNA was more abundant in the IPC group compared to the PN group at 24–72 h (*P*<0.05). For IGF-1 mRNA, however, there were no statistically significant differences between the three groups (Fig. 8).

### Angiogenic Factor Protein Expression

**Figure 9:**
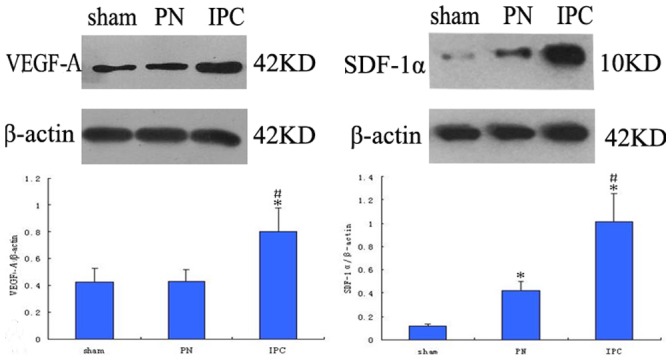
Relative expression of VEGF-A (6 h) and SDF-1α (24 h) protein. Protein expression was assessed by Western blot analyses using β-actin as a sample loading control. VEGF-A level was significantly higher in the IPC rats compared with that in the PN or Sham group (*P*<0.05). However, there were no significant differences between VEGF-A levels in the PN and Sham groups. Although SDF-1α expression was significantly increased in the PN group when compared to the Sham group, the IPC group showed a greater increase in SDF-1α expression when compared to the PN group. Data are shown as mean ± SEM. *Significant difference vs. Sham group (*P*<0.05); ^#^significant difference vs. PN group (*P*<0.05).

VEGF-A, SDF-1α, and IGF-1 protein expression were also examined. As shown in Fig. 9, VEGF-A expression in the IPC group was significantly increased compared with the PN and Sham groups at 6 h (*P*<0.05). However, there was no difference between VEGF-A expression in the PN and Sham groups. SDF-1α protein was expressed at higher levels in the PN and IPC groups compared with the Sham group at 24 h; the IPC group showed a greater increase in SDF-1α expression when compared to the PN group (*P*<0.05). For IGF-1 expression, however, there was no significant difference between groups.

## Discussion

PN is more frequently applied in urology, particularly to treat renal cell carcinoma (RCC). The main advantage of PN includes maximal preservation of renal parenchyma, which helps to avoid end-stage renal disease [19], [20]. There was no clear evidence that PN was associated with an inferior oncological outcome with stage T1a-T1b or even T2 cancer [4], [5]. In addition, RN may impact long-term survival compared with PN for renal tumors, the former being associated with increased risks of cardiovascular morbidity [21]. Unfortunately, PN is associated with kidney IRI related to renal pedicle clamping during surgery, which has potentially detrimental effects on subsequent renal function and survival. Multiple studies have demonstrated that IPC plays a protective role in a variety of organs including the kidneys [14], [22], however the protective mechanisms of renal IPC remain unclear. Hence, the present study established a renal IPC model and demonstrated that the early phase of IPC increases the number of EPCs in the ischemic kidney, thereby alleviating kidney injury and preserving renal function.

There are different protocols in the model of kidney IPC, and there was no consensus about the critical threshold of protection in the kidney [22], [23]. In this study, the preconditioning scheme of Torras *et al.* [24] and Jia *et al.* [25] was adapted to create a rat model of renal IRI. We demonstrated that one cycle of 15 min of ischemia and 10 min of reperfusion significantly attenuated renal tubular disruption and reduced kidney dysfunction caused by 40 min of artery blockage. It is worth mentioning that previous studies have indicated that the protective effects of the early phase of IPC only lasted for minutes to hours [26]. In the present study, however, we found that it afforded a longer duration of renoprotection (three days).

Several mechanisms could play a role in the protection afforded by IPC. Previous studies demonstrated that renal protection by IPC was associated with inhibition of NF-κB activation [27] or with formation of p50/p50 homodimers [28]. Other studies showed that IPC increases nitric oxide production, which has a protective effect against IRI [29]. Furthermore, recent studies showed that IPC participates in stem cell mobilization and the latter was closely related to ischemic repair [30], [31]. These findings suggest that increased numbers of EPCs may offer a possible explanation for the observed protective effects of IPC. Studies by Patschan and colleagues [13] showed that the late phase of IPC facilitates EPC mobilization to the ischemic kidney, and accumulation of EPCs in the kidney is at least partially responsible for the beneficial effects of IPC. However, the interval between pre-ischemia and ischemic injury is too long for a clinical application of the protocol. In the present study, the early phase of IPC significantly increased the number of EPCs in the ischemic kidney and afforded partial renoprotection following PN. These findings provide evidence for EPCs modulation by the early phase of IPC, which attenuates IRI, and are in agreement with the reports of Li *et al.* [14] who stated that acute myocardial ischemia may be alleviated by EPC recruitment during the early phase of IPC.

Renal IRI refers to a complex disorder that comprises multiple causative factors [32]. Tubular epithelial cells dedifferentiate, proliferate and replace the injured epithelial cells during recovery from IRI; loss of tubular epithelial cells is of key importance for the pathophysiological consequences of the syndrome [33], [34]. Recent studies also found that endothelial cells in peritubular capillaries play an important role in renal IRI, where there is swelling, blebbing, death, and detachment of viable cells, leading to impairment of the microcirculation following IRI. These phenomenon, described as “no-reflow,” were proposed to be responsible for a delayed functional recovery of the post-ischemic kidney [32], [35]. Furthermore, infusion of endothelial cells into rats subjected to renal artery clamping led to improvement of renal microcirculation and mitigation of the organ dysfunction [36]. It is encouraging that EPCs play a fundamental role in cell regeneration and vascular repair [9]. The present study showed that the number of EPCs in the kidneys is modulated by IPC and promotes proliferation of endothelial and epithelial cells one day following surgery. This was demonstrated using immunochemistry, which detected a large number of PCNA^+^ cells in the medullopapillary region. In addition, there was a significant increase in angiogenesis with preconditioned rats, as measured by PCRI. EPCs at least partially participate in neovascularization and cell regeneration that may be critical to recovery from ischemic injury.

The mechanisms could be attributed to incorporation into the injured cells and paracrine effects [37]–[40]. Previous studies showed that only low numbers of EPCs could be identified as incorporating into the new capillaries following EPC transplantation, suggesting that EPCs do not act via direct incorporation into the injured cells, but rather by a paracrine mechanism [41], [42]. Previous studies confirmed that EPCs have the ability to secrete VEGF, SDF-1α, granulocyte colony stimulating factor (G-CSF), granulocyte macrophage colony stimulating factor (GM-CSF), interleukin-8 (IL-8), IGF-1, hepatocyte growth factor (HGF), and transforming growth factor-beta1 (TGF-β1), each playing different functions in tissue repair and reconstruction [43]. Interestingly, paracrine factors greatly increase EPC-mediated angiogenesis [44], [45] and play an important role in mobilization, migration, homing, and differentiation of EPCs [46], [47]. In the present study, VEGF-A and SDF-1α expression was significantly increased in the IPC group, which may explain the kidney-protective functions through paracrine effects.

There were also a few limitations in this study. First, there are certainly several factors that can affect the capacity of IPC in renal protection, and EPCs are only one such factor. As the observations were phenomenological and no cytological experiments were conducted, it is difficult to attribute all of the protective benefit to EPCs. Second, there was no long-term observation of the effects of IPC on PN. Thus, further experimental data need to be provided to substantiate a causal mechanism and to observe the effects of IPC on PN for longer time periods.

In conclusion, the early phase of IPC increases the number of EPCs in the kidney medullopapillary region, which affords partial renoprotection following PN, suggesting the role of EPCs in functional rescue. The protective effects of EPCs were associated with secretion of angiogenic factors, which could promote proliferation of endothelial and epithelial cells as well as angiogenesis in peritubular capillaries. It is proposed that IPC should be provided before PN to ameliorate the potential renal IRI.
